# Biocontrol of *Candida albicans* by Antagonistic Microorganisms and Bioactive Compounds

**DOI:** 10.3390/antibiotics11091238

**Published:** 2022-09-12

**Authors:** Honghua Li, Jinpeng Yang, Xinwan Zhang, Xiuli Xu, Fuhang Song, Hehe Li

**Affiliations:** 1 School of Light Industry, Beijing Technology and Business University, Beijing 100048, China; 2 School of Ocean Sciences, China University of Geosciences, Beijing 100083, China; 3 Key Laboratory of Brewing Molecular Engineering of China Light Industry, Beijing Technology and Business University, Beijing 100048, China

**Keywords:** *Candida albicans*, antagonistic microorganisms, biocontrol strategy, bioactive compounds

## Abstract

*Candida albicans* is an endogenous opportunistic pathogenic fungus that is harmless when the host system remains stable. However, *C. albicans* could seriously threaten human life and health when the body’s immune function declines or the normal flora is out of balance. Due to the increasing resistance of candidiasis to existing drugs, it is important to find new strategies to help treat this type of systemic fungal disease. Biological control is considered as a promising strategy which is more friendly and safer. In this review, we compare the bacteriostatic behavior of different antagonistic microorganisms (bacteria and fungi) against *C. albicans*. In addition, natural products with unique structures have attracted researchers’ attention. Therefore, the bioactive nature products produced by different microorganisms and their possible inhibitory mechanisms are also reviewed. The application of biological control strategies and the discovery of new compounds with antifungal activity will reduce the resistance of *C. albicans*, thereby promoting the development of novel diverse antifungal drugs.

## 1. Introduction

Fungal infection is a common global problem affecting humans and its incidence is on the rise [1]. Among them, *Candida* has been a life-threatening pathogen for a long time, accounting for almost 80% of fungal infections. Recently *C. albicans* infection causes more than 400,000 cases of blood infection each year, with a mortality rate of about 42% [2,3,4]. *C. albicans*, a small number in the normal body, is a part of healthy flora. It can exist in the oral cavity, intestinal tract, upper respiratory tract, and other parts. When growing in the state of unicellular yeast cells, it does not cause disease. However, when the normal flora interacts with each other out of balance or the body’s immune function and defenses decline, *C. albicans* proliferates and grows into hyphae, invading cells and causing disease. It has been a major cause of morbidity and mortality in immunocompromised populations [5].

In host, the pathogenicity of *C. albicans* is caused by the decline of immune function, the change of conventional flora and the destruction of the epithelial protective barrier. During infection, the formation of *C. albicans* biofilm and the morphological switch from yeast-like to hyphal-like are considered to be two significant pathogenic characteristics of *C. albicans*. First of all, its morphological plasticity is crucial to the pathogenicity of fungi, as the hyphal form has a key role in the infection process [6,7,8]. In addition, the pathogenicity of *Candida* is greatly enhanced by the formation of biofilms [9]. Biofilms are microbial communities that irreversibly attach to surfaces. Biofilms behave very differently from planktonic cells, and once formed, they can increase resistance to existing antibiotics and immune responses [10]. Therefore, inhibition of hyphal development and inhibition of biofilm formation are considered to be an effective strategy against *C. albicans* infection.

Currently, there are very few drugs for the treatment and prevention of *Candidiasis* in clinic. The polyene antibiotic is the earliest specific drug isolated from *Streptomyces nodosus* in the 1950s to treat yeast infection. Since then, many antifungal agents have been developed [11,12]. There are four types of antifungal agents for *C. albicans* infection [13]. The most commonly used antifungal drugs and the mechanism of action include:(1) The widest range and most effective is polyene (Amphotericin B), which can kill most fungi. Polyenes bind to ergosterol in fungal cell membranes, creating stomata and causing cell death [14,15]. (2) Triazole antifungal drugs (fluconazole, voriconazole and itraconazole). Azoles can inhibit lanosterol 14α demethylase, which is an important enzyme in ergosterol biosynthesis [16,17,18,19]. (3) 5-fluorocytosine, it inhibits fungal DNA synthesis by inhibiting thymidylate synthetase [12,20]. (4) There are also some echinocandin antifungal drugs (anidulafungin, micafungin, and caspofungin) [21,22,23,24,25,26,27]. The mechanisms of these bioactive compounds against *C. albicans* are mainly related to inhibition of biofilm formation, inhibition of virulence factors and destruction of cell wall integrity. With the increasing drug resistance of *C. albicans*, it is compelling to find new antifungal methods and reagents to solve this complex medical problem. Biological control is considered to be a more effective and safe strategy [1,28,29].

Novel natural compounds produced by microorganisms, due to their complex structures, may exhibit novel antibacterial mechanisms and different modes of action. Moreover, they were considered as candidates to reduce drug resistance. People have been trying to find unique antifungal drugs from nature, which has led to important advances in the development of new antifungal drugs.

In recent years, there have been some reviews on natural products that could inhibit *C. albicans* [2,30,31,32,33,34,35,36,37]. In this paper, we have reviewed the antagonistic microorganisms against *C. albicans* considered in recent years and have also reviewed the active natural products produced by microorganisms that inhibit *C. albicans*. Researchers focus on the study of antagonistic microorganisms in order to use probiotics to inhibit *C. albicans*. Through the review of secondary metabolites, it can provide a reference for clinical drug development.

## 2. Antagonistic Microbes against *C. albicans*

Traditional azoles and their derivatives have poor effect on preventing recurrence of pathogenic fungus. In some patients, fluconazole can cause some side effects such as headache, discomfort, dizziness, gastrointestinal and, rash [38]. Bacteria, yeast, and fungus all can develop resistance to antibiotics and bactericidal chemicals [39]. Biological control of microbial infections is an alternative approach that utilizes antagonistic microorganisms to prevent the growth and infection of harmful microorganisms. Diverse microorganisms, including fungi (such as non-toxic *Aspergillus*, *Trichoderma*, *Penicillium*), yeast strains, and bacteria, have been studied as potential antagonistic organisms for the control of *C. albicans*. In this review, the microorganisms that inhibit *C. albicans* and their secondary metabolites are introduced from the perspective of antagonistic microorganisms. The microorganisms that have potential antagonism against *C. albicans* are listed in Table 1. The main species and inhibition activities of these antagonistic strains are also discussed. We have reviewed the antagonistic microorganisms against *C. albicans* in recent years with the aim to develop a new natural material, using beneficial bacteria or fungus, that would be useful for inhibiting the growth of pathogenic *C. albicans* in the human body.

**Figure 1:**
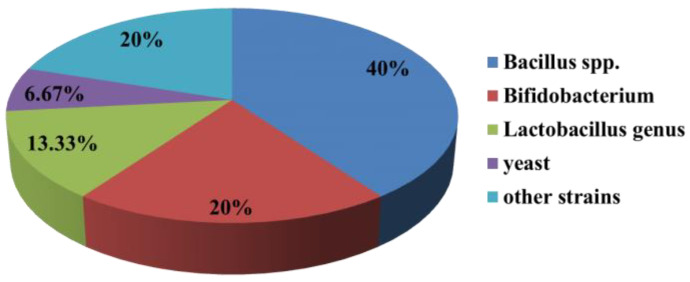
Percentages of different antagonistic microbes of *C. albicans*.

As shown in Figure 1, the article reporting *Bacillus* spp. antagonists were dominant (40%) compared with the article reporting antagonistic *Bifidobacterium* (20%), antagonistic *Lactobacillus genus* (13.33%), antagonistic yeast (6.67%) and other antagonistic strains (20%).

### 2.1. Antagonistic Effect of Bacillus *spp.* against C. albicans

Some beneficial bacteria or fungus are widely used in biocontrol. In particular, it is well known that *Bacillus* spp. is an excellent source of antifungal drugs, thus *Bacillus* spp. is widely used as a biological control agent [51,52,53]. Bacillus species are Gram-positive bacteria that can survive in different environments. They could form endospores and produce a large number of metabolites [53].

Researchers isolated four strains of *Bacillus* A16 (*B. sphaericus*), M142 (*B. circulans*), M166 (*B. brevis*) and T122 (*B. brevis*) from soil samples. These *Bacillus* showed extensive inhibitory activity against *C. albicans* [40]. Among them, *B. brevis* M166 showed antifungal activity against all tested microorganisms (*Sclerotium rolfsii*, *Rhizoctonia solani*, *Fusarium oxysporum*, *Staphylococcus aureus* and *C. albicans*), with a relatively wide antimicrobial spectrum. *B. circulans* M142 had strong antibacterial activity against *C. albicans* and *S. aureus*, while *B. brevis* T122 only had antibacterial activity against *C. albicans*. To our knowledge, no specific compounds inhibiting *C. albicans* had been identified.

In addition to the antifungal activity of *Bacillus* spp. from soil samples, *Bacillus* spp. from marine samples was also found to have inhibitory activity against *C. albicans. B. subtilis spizizenii* DK1-SA11 was isolated from Bay of Yellow Sea in China [41]. The cell-free supernatant had significant inhibitory activity against *C. albicans*. The inhibitory active ingredient had not been identified but was stable in nature, while the enzymatic hydrolysis of lipase, trypsin and papain made it lose activity. Antimicrobial activity tests against pathogens indicated that this strain could be used as a source of antibiotics, synbiotics, and probiotics. 

*B. velezensis* was widespread in the environments and produced abundant lipopeptides with good bacteriostatic effect. Some researchers have studied on the inhibitory spectrum of *B. velezensis* DTU001 against 20 different species of human and/or plant pathogenic fungi [42]. The results showed that *B. velezensis* DTU001 was superior to a single lipopeptide (fengycin and iturin) in inhibiting the selected fungi. Co-culture of *B. velezensis* DTU001 and *C. albicans* significantly inhibited *C. albicans* proliferation, which further supported the biological control properties of *B. velezensis* DTU001. 

*B. amyloliquefaciens* SYBC H47 was isolated from honey [43]. The cultured cell-free supernatant had significant inhibitory activity against *C. albicans*. The main antibacterial substances were surfactin, fengycin and bacillomycin. Three compounds had an inhibitory effect on spore germination of *Botryosphaeria dothidea*. However, compounds that inhibit *C. albicans* had not been identified. 

*Bacillus velezensis* 1B-23 had inhibitory effect on the growth of *C. albicans* in vitro. It had a certain application prospect as a biological agent for biological control of fungal pathogens [44].

### 2.2. Antagonistic Effect of Bifidobacterium *spp.* against C. albicans

*Bacillus* spp. has been used clinically because of its bacteriostatic activity. Another probiotic, *Bifidobacterium*, can also be used to prevent and treat intestinal flora disorders in clinic. *Bifidobacterium* is a vital member of the normal human gut microbiota. Some strains of *Bifidobacterium* can be used as probiotics in food, medicine and feed [54,55]. *Bifidobacteria* could produce acetic acid and/or lactic acid during metabolism. Moreover, the action of lactic acid would reduce intestinal pH. Thereby, *Bifidobacterium* could inhibit the proliferation of pathogenic microorganisms [56,57]. 

*Bifidobacterium longum* BB536 which was isolated from the feces of healthy infants had been commercially used in various food applications and was considered safe [45,46,58]. The researchers fermented broccoli using *B. longum*. The supernatant could inhibit the growth of *C. albicans* and some other pathogenic bacteria in vitro. Researchers used beneficial bacteria such as *bifidobacteria* and used broccoli as a substrate for the growth of beneficial bacteria to develop substances. Maybe, we can use beneficial microorganisms and their secondary metabolites to develop products that inhibit the growth of pathogenic microorganisms. For example, as a daily oral care preparation, it can prevent the growth of *C. albicans* in human oral cavity [59].

### 2.3. Antagonistic Effect of Lactobacillus *spp.* against C. albicans

*Lactobacillus johnsonii* is a probiotic with wide antimicrobial characteristics and can be used as an antiallergic drug. Recent studies have shown that *L. johnsonii* also has inhibitory effects on *C. albicans. L. johnsonii* MT4 was isolated from the oral cavity of healthy mice. The strain affected the *C. albicans* growth in both biofilm and planktonic conditions. *L. johnsonii* MT4 showed an antagonistic effect on *C. albicans*, thus inhibiting the biofilm formation of *C. albicans* and planktonic growth. The study on the strain genome had shown that it produced metabolites with anti-*C. albicans* activity, but no active substances against fungi have been reported so far. The antibacterial mechanism needed to be further explored [47].

In addition to producing secondary metabolites that antagonize *C. albicans*, the competition for ecological niches of different strains during the growth process would also cause antagonism among strains, such as *C. albicans* and lactic acid bacteria in the gastrointestinal (GI) tract [48,60]. Non-pathogenic colonization of the human GI tract by *C. albicans* was common. *C. albicans* could regulate bacterial community in mice treated with broad-spectrum antibiotics. One of the most striking features was the significant change in the lactic acid bacteria (LAB) levels. *C. albicans* and *Lactobacillus* species shared a metabolic niche throughout the GI tract. LAB could antagonize *Enterococcus* and *C. albicans* in the GI tract. *C. albicans* and *Lactobacillus* could mutually regulate each other’s growth and virulence in the GI tract [48].

### 2.4. Antagonistic Effect of Yeast against C. albicans

In addition to the bacteria mentioned above, yeast can also be used for biological control. *Metschnikowia* could accumulate pigments in cells and growth media. It was a highly effective biocontrol yeast. Antagonism of *M. pulcherrima* against phytopathogens had been demonstrated [49]. The researcher investigated three new strains of *Metschnikowia* which were isolated from grapes. The strain had strong antagonistic activity against *C. albicans*. The three strains produced the same amount of nevus pigments, but there were significant differences in antifungal activities against different microorganisms [61,62].

### 2.5. Antagonistic Effect of Other Strains against C. albicans

*Salivarius* MG242 isolated from human vagina presented a potential application in the biological control of *C. albicans*. MG242 had an obvious inhibitory impact on *C. albicans*, and the strain had the possibility to be developed into a probiotic product for the treatment of *C. albicans*. In order to develop stable living cell products, it was necessary to maintain anti-*Candida* activity and preserve cell viability during lyophilization. Lower storage temperature extended shelf life to 8.31 months [50]. Strains of K124 (*P. fluorescens*) was also isolated from soil samples, e.g., *B. sphaericus* A16, *B. circulans* M142, *B. brevis* M166 and *B. brevis* T122. *P. fluorescens* K124 showed extensive inhibitory activity against *C. albicans* [40]. *P. fluorescens* K124 only had antifungal activity against *C. albicans*. At present, no inhibitory compounds produced by the strain have been identified.

### 2.6. A Conclusion of Antagonistic Microbes

In conclusion, *Bacillus*, *Bifidobacterium*, *Lactobacillus*, and yeast strains can antagonize the growth of *C. albicans*. In particular, many strains of *Bacillus* have obvious advantages to exert antagonistic strains. Most of the strains exert antagonistic effects by producing active compounds. Moreover, some inhibit the growth of *C. albicans* through niche competition. We should intensify research on strains with inhibitory activity, especially probiotics. Research on different strains, especially probiotics, with antifungal activity is helpful to develop the agent for inhibiting *C. albicans*. Since the effective components of some strains against *C. albicans* are not clear, the compounds with obvious inhibitory activity should be further analyzed.

## 3. Inhibitory Nature Metabolites Produced by Diverse Antagonists

Secondary metabolites derived from many plants and microorganisms are valuable natural compounds. Many natural products have significant biological activities, such as anti-tumor activity, antibacterial activity [63,64,65]. The antagonistic effect of the strain is mainly due to the production of natural secondary metabolites, such as antibiotics and antimicrobial peptides [66,67,68]. The antifungal compounds reviewed in this paper are secondary metabolites derived from microorganisms for biological control of *C. albicans* and have strong inhibition against *C. albicans*. Table 2 lists the various antagonistic microbial strains, the characteristics of the active compounds produced, and their inhibition mechanism against *C. albicans*. Table 3 lists the structure and the activity of these inhibitory compounds.

### 3.1. Nature Products Produced by Bacteria

Bacillus produces diverse active compounds, such as proteases, amylases, surfactants, and antibiotics [66,96,97,98,99]. Due to the high yield of antifungal active substances and the advantage of releasing peptides directly into the extracellular, *Bacillus subtilis* is a potential strain for the production of antifungal compounds [100,101,102]. The *B.subtilis* isolated from marine had antifungal membrane effect on *C. albicans*. It was found that 5-hydroxymethyl-2-furaldehyde (5HM2F) was one of the main components that inhibited *C. albicans* in the fermentation broth [69]. 5HM2F effectively disrupted the hyphal-like morphological transition of *C. albicans* and prevented the initial adhesion process. Further studies showed that 5HM2F reduced the main source of biofilms by reducing the levels of secreted virulence factors and ergosterol. In addition, the combination of 5HM2F with azole antifungal drugs effectively enhanced the anti-*C. albicans* activity of the tested drugs. Transcriptional level studies showed that 5HM2F increased the sensitivity of *C. albicans* to antifungal drugs by negatively regulating the expression levels of genes related to drug resistance mechanisms. As an antagonist, 5HM2F effectively inhibited the biofilm formation and reduced the resistance of *C. albicans* to traditional antifungal drugs.

*Pantoea agglomerans* are widespread in the environment [103,104]. *P. agglamerans* strain C9-1 was used as a biological control agent (BlightBan C9-1). A peptide antibiotic was isolated. The compound was 2-amino-3-(oxane-2,3-dicarboxamido)propanoyl-valine. This compound showed effectively inhibition on the growth of *C. albicans* [70].

Six novel alkaloids containing phenethylamine (PEA) were isolated from the culture medium of *Tenacibaculum* *discolor* sv11. Among them, Dipyrrolepyridines A and B had certain inhibitory activity against *C. albicans* FH2173 [71].

### 3.2. Nature Products Produced by Yeast

The researchers found that *S*. *boulardii* had inhibitory activity on *C. albicans*. The fermentation broth extracts inhibited hyphae formation, adhesion and biofilm development of *C. albicans* [72]. Further analysis showed that the fermentation broth contained 2-phenylethanol, capric, caprylic and caproic acid. The fermentation broth and the isolated pure compounds were tested for biological activity against *C. albicans*. Capric acid inhibited hyphae formation in *C. albicans* and also reduced adhesion and biofilm formation. However, compared with *S. boulardii* extract, the inhibitory effect on *C. albicans* was reduced by three times in the case of capric acid alone, so other compounds were contained to inhibit the adhesion of *C. albicans*. The transcriptional levels of *CSH1*, *INO1*, and *HWP1* genes were decreased in *C. albicans* treated with *B. boulardii* extract and capric acid.

### 3.3. Nature Products Produced by Endophytic Fungi

Biatriosporin D (BD), A phenolic compound, was isolated from *Biatriospora* spp. [73]. The compound inhibited biofilm formation, hyphal morphogenesis and adhesion of *C. albicans*. Notably, BD efficiently inhibited hyphal formation at doses lower than MIC value. Further studies showed that BD regulated the Ras1-cAMP-Efg1 pathway through reducing the cAMP level. As a prodrug, BD showed potential action against *C. albicans*. This provided possible application prospects for BD against clinically opportunistic fungi by targeting fungal virulence.

A fungus *Drechmeria* sp. was isolated from the roots of Panax notoginseng. Four known analogs and seven new indole diterpenoids, drechmerins A-G, were isolated from the fermentation broth. The MIC value of Drechmerin B against *C. albicans* was 12.5 μg/mL [74].

Five new polyketides and four known analogs were isolated from the *Phoma* sp. SYSU-SK-7 [75]. Among them, the polyketide colletotric B had strong antifungal activity against *C. albicans*, and the MIC value of colletotric A was 3.27 μg/mL. The MIC value of 3-hydroxy-5-methoxy-2, 4, 6-trimethylbenzoic acid was 2.62 μg/mL, and the MIC value of orsellinic acid was 2.10 μg/mL.

Three new monomers were isolated from the marine strain *Stachybotrys* *Chartarum*. The MIC value of compound Atranone Q was 8 μg/mL [76].

Nine sesquiterpenes and three diterpenes were isolated from the fermentation broth of the *Xylaria* sp. YM 311647 [77]. The MIC values of nine sesquiterpenes against *C. albicans* were different, while the activity of diterpenes was higher. One of the sesquiterpenes had the highest antibacterial activity against *C. albicans*, with an MIC value 16 μg/mL.

### 3.4. Nature Products Produced by Marine Fungi

One of the prenylated indole alkaloids, waikialoid A, was isolated from a metabolite-rich *Aspergillus* strain near Waikiki Beach. IC_50_ value of the natural product was 1.4 μM in inhibiting biofilm formation. Another compound, waikialide A, could inhibit the formation of *C. albicans* biofilm with a weaker IC_50_ value of 32.4 μM [78].

Two new 13-membered macrolide compounds and known PF1163A, B, D, H and F were isolated from *penicillium* strain. All of them had inhibitory activity against *C. albicans* when used in conjunction with fluconazole [79].

Three drimane sesquiterpene purpurides E-G were isolated from *P. minioluteum* ZZ1657. Purpurides E exhibited inhibitory activity against *C. albicans* with MIC values of 6–12 μg/mL, and Purpurides F was 3–6 μg/mL [80].

### 3.5. Nature Products Produced by Marine Source Actinomycetes

One new phenylpyridinealkaloid, five known analogues and five new bipyridine alkaloids were isolated from *Actinoalloteichus cyanogriseus* WH1-2216-6. The MICs of caerulomycin A and C against *C. albicans* were 21.8 and 19.3 μM, respectively [81].

Two new 36-membered macrolides, Bahamaolides A and B, were isolated from sediments of marine actinomycetes (*Streptomyces* sp.) on the North Cat Reef, Bahamas. Bahamaolides A obviously inhibited isocitrate lyase of *C. albicans* [82].

Streptovitacin A and new Streptoglutarimides A-J were isolated from marine actinomycetes *Streptomyces* sp. ZZ741. The MIC values of the obtained compounds against *C. albicans* were 8–20 μg/mL, and Streptoglutarimides D had a better inhibitory effect with 8 μg/mL [83].

### 3.6. Nature Products Produced by Lichen

Usnic acid, a secondary metabolite of lichens, effectively inhibited the hyphal switching of *C. albicans*. Usnic acid significantly reduced the thickness of mature biofilms and prevented the adhesion of biofilms. At the biofilm inhibitory concentration (BIC), the inhibitory effect of usnic acid on *C. albicans* biofilm could reach 65% [84].

As an inhibitor, Retigeric acid B (RAB) derived from *lichen* significantly inhibited the hyphae formation of *C. albicans* [105,106,107]. RAB prolonged the survival time of nematodes infected by *C. albicans*. RAB regulated the Ras1-CAMP-Efg1 pathway by reducing cAMP levels and inhibitd hyphal formation. By inhibiting the interruption of yeast-hyphal morphological transition and weakening the virulence of *C. albicans*, it provided a potential application for the treatment of *C. albicans* infection [85].

Funiculosone, a substituted dihydroxanthene-1, 9-dione, was isolated from the lichens of the *Trichosporaceae* fungus *T. funiculosus*. The IC_50_ value of *T. funiculosus* was 35 μg/mL [86].

### 3.7. Nature Products Produced by Other Fungal Sources

8-deoxytrichothecin and trichodermol, isolated from the *Acremonium* sp. PSU-MA70, exhibited moderate antifungal activity against *C. albicans* [87]. Two compounds cyschalasins A and B were isolated from *Aspergillus Micronesiensis* and showed antifungal activity against *C. albicans* [88]. Moriniafungins B-G, a new tetracyclic diterpene glycoside of Sordarincin, was isolated from *Curvularia hawaiiensis* TA26-15. Moriniafungins B-G had antifungal activity against *C. albicans* with an MIC value of 2.9 μM [89].

The F2 mycotoxin zearalenone (ZEN) produced by *Fusarium* and *Gibberella* species exhibited in vitro inhibitory effects on different microbial strains [90,91]. 100 μg/mL ZEN treatment significantly inhibited *C. albicans* hyphal morphogenesis and biofilm formation. Similarly, ZEN effectively destroyed established *C. albicans* biofilms without disturbing the planktonic cells. In vivo, ZEN prominently inhibited *C. albicans* infection in *Caenorhabditis elegans* [92].

Deoxynivalenol (DON), produced by *Fusarium* spp., was an epoxide sesquiterpene compound [93,108,109,110]. DON and 3-acetyl-DON exhibited a dose-dependent inhibitory effect on *C. albicans* in vitro. DON obviously reduced *C. albicans* metabolic activity, disrupted pre-formed biofilms, inhibited biofilm formation and inhibited hyphal that embedded in free-living planktonic cells and colonies. DON and 3-acetyl-DON mimicked the mechanism of through interplaying with lanosterol 14α-demethylase that was like the action of azole drugs. DON exhibited antifungal filament and antifungal membrane potential against *C. albicans* [111].

A carefully scheduled fermentation of *P. camembertii/clavigerum* and *P. fuscum* yielded eight novel 16-membered ring macrolides, Berkelilactone A exhibited the most potent antifungal activity in the macrolide series. It had low micromolar inhibitory activity against *C. albicans* (MIC = 1–2 μg/mL). Berkelilactone A did not inhibit protein synthesis and did not target ribosomes, suggesting a new mode of mechanism for its antibiotic activity, but the specific mechanism had not yet been elucidated [94].

*U. maydis* secreted a large amount of the glycolipid biosurfactant ustilagic acid. The new glycolipid ustilagic acid C and B were induced under special culture conditions. And the two compounds showed weak antifungal activity against *C. albicans* [95].

### 3.8. A Conclusion of Inhibitory Compounds Produced by Antagonistic Microbes

Many natural products that obtained from diverse microbial sources have been successfully applied in many fields. To overcome the increasing drug resistance of *C. albicans*, the discovery of new natural antifungal compounds is necessary. This review summarizes about 30 different compounds produced by microorganisms that have been found to have inhibitory effects on *C. albicans*. These compounds are derived from different bacteria and fungi, including bacteria such as *Bacillus*, *T. discolor* sv11 and *P. agglomerans*; yeast such as *S. bombicola* and *S. boulardii*; *Phoma* spp. SYSU-SK-7, *Biatriospora* sp.; marine-derived fungi such as *Aspergillus*, *P. minioluteum* ZZ1657; *Streptomyces* sp.; *A. cyanogriseus* WH1-2216-6; *Streptomyces* sp. ZZ741 and *Actinomycetes* of marine origin; other fungal sources: *Fusarium*, *Gibberella* species, *P. brown, P. camembertii/clavigerum*, *C. Hawaiian ensis* TA26-15, *U. maydis*; *A. micronesiensis, Acremonium* sp. PSU-MA70 and other fungi. It can be seen from Table 2 that the antifungal mechanisms of most isolated known or unknown compounds have not been clearly analyzed. Only a few compounds have been studied at the transcriptional level. These microorganisms produce compounds with different structures to inhibit *C. albicans* in different ways, such as inhibiting biofilm formation and hyphal morphological transformation.

## 4. Conclusions

With the emergence of *C. albicans* resistance against conventional antifungal therapies, new strategies to treat *C. albicans* infection are important [112]. Considering that *C*. *albicans* could threaten human life and health when the body’s immune function declines or the normal flora is out of balance. both *Bacillus licheniformis* and *Bifidobacterium* can be used in clinic to prevent and treat intestinal microbiota disorders. This article reviews the different antagonistic microorganisms of *C. albicans* and various bioactive secondary metabolites produced by microorganisms, which are expected to achieve biological control of human pathogenic fungus *C. albicans*.

Biological control of microbial infections is an alternative approach that utilizes antagonistic microorganisms to prevent the growth and infection of harmful microorganisms. Antagonistic microbes, such as bacteria, yeast, and fungus, have been studied as potential antagonistic organisms for the control of *C. albicans*. Through the study on diverse strains with antifungal activity, it is helpful to develop the agent for inhibiting *C. albicans*. This is a potential strategy for biological control of *C. albicans*. On the other hand, secondary metabolites derived from microorganisms are valuable natural compounds. Many natural products have diverse structures and can exhibit significant biological activities. The structures of these compounds include: macrolides, terpenoids, alkaloids, organic acids, and other heterocyclic compounds. The secondary metabolites introduced in Table 2 and Table 3 can significantly inhibit *C. albicans*. They are produced by diverse microorganisms. However no identified compounds are currently used as a drug against *C. albicans*. There are still four types of antifungal agents for *C. albicans* infection: polyene, triazole, 5-fluorocytosine, and echinocandin antifungal drugs [13]. Through the study of these active compounds, it is expected to obtain new drugs for the treatment and prevention of *C. albicans* infection, thereby maintaining human health.

## Figures and Tables

**Table 1:** Antagonistic Microbes against *C. albicans*.

Antagonists	Species	Activity	References
*Bacillus* spp.	*B. sphaericus* A16, *B. circulans* M142, *B. brevis* M166, *B. brevis* T122	Strains showed extensive inhibition against *C. albicans*.	[40]
	*B. subtilis spizizenii* DK1-SA11	Cell-free supernatant had significant inhibitory activity against *C. albicans*.	[41]
	*B. velezensis* DTU001	Significantly inhibited the proliferation of *C. albicans*, and the inhibition ability of the strain was better than that of a single lipopeptide.	[42]
*Bifidobacterium*	*B. amyloliquefaciens* SYBC H47	Cell-free supernatant and Cell suspension had obvious inhibition against *C. albicans*.	[43]
	*B. velezensis* 1B-23	Inhibited *C. albicans* growth in vitro.	[44]
	*B. longum* BB536	The supernatant of fermented broccoli could inhibit the growth of *C. albicans* in vitro.	[45,46]
*Lactobacillus genus*	*L. johnsonii* MT4	Inhibited planktonic growth and biofilm formation of *C. albicans*	[47]
	*Lactobacillus*	Regulated growth and virulence of *C. albicans* through niche competition.	[48]
Yeast	*Metschnikowia pulcherrima*	Strong antagonistic activity against *C. albicans*.	[49]
Other strains	*Enterococcus*	Regulated growth and virulence of *C. albicans* through niche competition.	[48]
	*Pseudomonas fluorescens*	The strain showed extensive inhibition against *C. albicans*.	[40]
	*Salivarius* MG242	The strain had significant inhibitory effect on *C. albicans*.	[50]

**Table 2:** Inhibitory nature metabolites produced by antagonists against *C. albicans*.

Sources	Inhibitory Compounds	Main Characteristics of the Compounds	Other Inhibitory Actions	References
Bacteria				
*Bacillus subtilis*	5HM2F	Inhibit morphological transition	Reduced levels of secreted virulence factors and ergosterol to reduce the main sources of biofilms.	[69]
*Pantoea agglomerans* C9-1	2-amino-3-(oxane-2,3-dicarboxamido) propanoyl-valine	Inhibit growth	None	[70]
*Tenacibaculum* *discolor* sv11	Dipyrrolepyridines A and B	Inhibit growth		[71]
Yeast				
*Saccharomyces* *boulardii*	Capric acid	Inhibit hyphal formation, adhesion and biofilm development	Transcriptional levels of *HWP1*, *INO1* and *CSH1* genes were decreased.	[72]
Eendophytic fungi				
*Biatriospora* sp.	Biatriosporin D	Inhibit adhesion, biofilm formation and hyphal morphogenesis	Regulated Ras1-CAMP-Efg1 pathway, disrupted morphological transition and attenuated virulence	[73]
*Drechmeria* sp.	Drechmerin B	Inhibit growth	None	[74]
*Phoma* sp. SYSU-SK-7	Colletotric A	Inhibit growth	None	[75]
*Stachybotrys* *chartarum*	Atranone Q	Inhibit growth	None	[76]
*Xylaria* sp. YM 311647	Sesquiterpenes and Isomatanic diterpenes	Inhibit growth	None	[77]
Marine fungi				
*Aspergillus* isolates from Waikiki Beach	Waikialoid A and Waikialide A	Inhibit biofilm formation	None	[78]
*Penicillium meleagrinum* var. *viridiflavum*	PF1163A and B	Inhibit growth	None	[79]
*Penicillium minioluteum* ZZ1657	Purpurides E and F	Inhibit growth	None	[80]
Marine actinomycetes				
*Actinoalloteichus cyanogriseus* WH1-2216-6	Caerulomycin A and C	Inhibit growth	None	[81]
*Streptomyces* sp.	Bahamaolides A	Inhibit isocitrate lyase	None	[82]
*Streptomyces* sp. ZZ741	Streptoglutarimides A-J and Streptovitacin A	Inhibit growth	None	[83]
Lichen				
lichens	Usnic acid	Reduce the thickness of mature biofilms and Inhibit biofilm adhesion.		[84]
lichens	Retigeric acid B	Inhibit hyphal formation	RAB regulated the Ras1-cAMP-Efg1 pathway and inhibited hyphal formation	[85]
Lichens with *Talaromyces funiculosu*	Funiculosone	Inhibit growth	None	[86]
Other strains				
*Acremonium* sp. PSU-MA70	8-Deoxytrichocin and trichodermol	Inhibit growth	None	[87]
*Aspergillus micronesiensis*	Cyschalasins A and B	Inhibit growth	None	[88]
*Curvularia hawaiiensis* TA26-15	Moriniafungins B-G	Inhibit growth	None	[89]
*Fusarium* and *Gibberella* species	Zearalenone	Inhibit biofilm formation of and hyphal morphogenesis	None	[90,91,92]
*Fusarium* spp.	Deoxynivalenol	Inhibit biofilm formation and reduce metabolic activity	DON and its derivatives interplayed with lanosterol 14a-demethylase	[93]
*Penicillium fuscum* and *Penicillium camembertii/clavigerum*	Berkleyolactone A	Inhibit growth	A new mode of action that had not been resolved	[94]
*Ustilago maydis*	Ustilagic acid B and C	Inhibit growth	None	[95]

5HM2F: 5-hydroxymethyl-2-furaldehyde.

**Table 3:** The structures and activity of compounds against *C. albicans*.

Inhibitory Compounds	Compound Structure	Activity	References
Terpenoids			
Isomatanic diterpenes	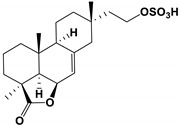	The MIC value was 16 μg/mL	[77]
Purpurides E and F	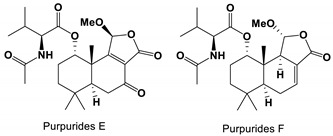	The MIC values were 12 and 6 μg/mL, respectively.	[80]
Usnic acid	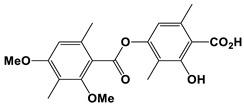	The MBIC value was 100 μg/mL.	[84]
Moriniafungins E	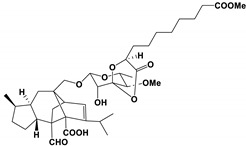	The MIC value was 2.9 μM.	[89]
Macrolides			
PF1163 A and B	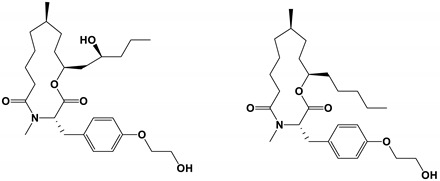	The inhibitory concentrations were 1 and 2 μg/mL, respectively.	[79]
Bahamaolides A	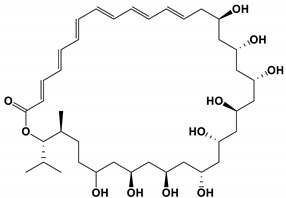	The MIC value was 12.5 μg/mL.	[82]
Berkleyolactone A	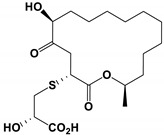	The MIC value was 1–2 μg/mL.	[94]
Organic acids			
Capric acid	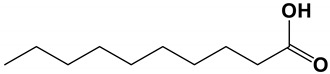	The inhibitory concentration was 45.3 μg/mL.	[72]
Retigeric acid B	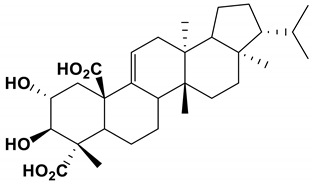	The MIC_80_ value was 8 μg/mL.	[85]
Ustilagic acid B and C	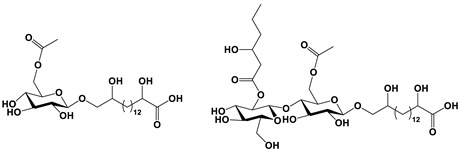	The MIC values were 50 and 100 μg/mL, respectively.	[95]
Alkaloids			
Ketones			
Colletotric A	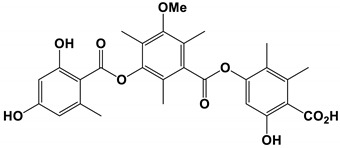	The MIC value was 3.27 μg/mL.	[75]
Atranone Q	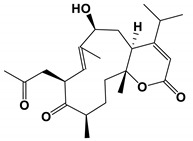	The MIC value was 8 μg/mL	[76]
Waikialoid A and Waikialide A	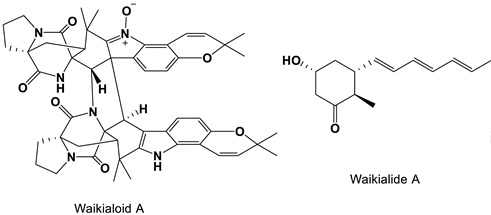	The IC_50_ values were 1.4 and 32.4 μM, respectively.	[78]
Caerulomycin A and C	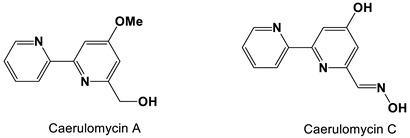	The MIC values were 21.8 and 19.3 μM, respectively.	[81]
Cyschalasins A and B	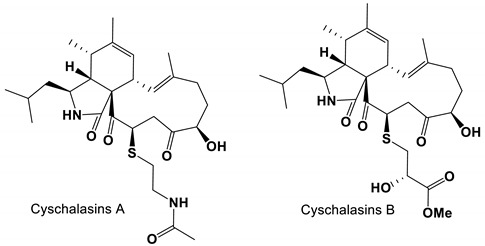	The MIC_50_ values were 43.3 ± 1.5 and 94.7 ± 1.3 μg/mL, respectively.	[88]
Zearalenone	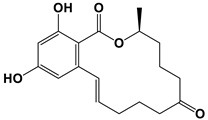	The inhibitory concentration was 100 μg/mL	[90,91,92]
Alcohols			
8-Deoxytrichothecin and trichodermol	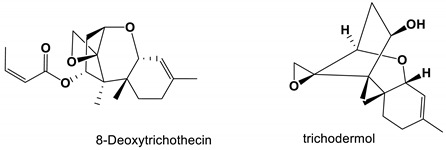	The MIC values were 16 and 64 μg/mL, respectively.	[87]
Deoxynivalenol and 3-acetyl-DON	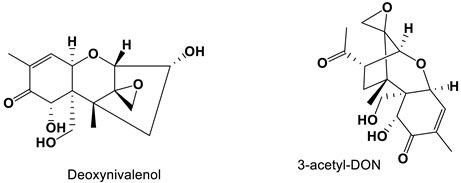	All inhibitory concentrations were 50 μg/mL.	[93]
Other structural compounds			
5HM2F	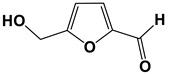	The MBIC value was 400 μg/mL.	[69]
2-amino-3-(oxane-2,3-dicarboxamido) propanoyl-valine	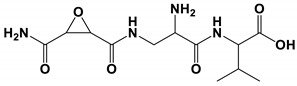	The inhibitory concentration was 1.5 μg/mL.	[70]
Dipyrrolepyridines A and B	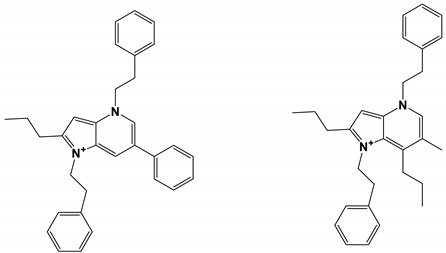	Certain antibacterial activity.	[71]
Biatriosporin D	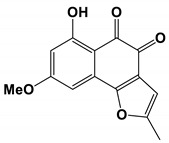	The inhibitory concentration was 2 μg/mL	[73]
Drechmerin B	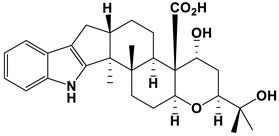	The MIC value was 12.5 μg/mL.	[74]
Streptoglutarimides D	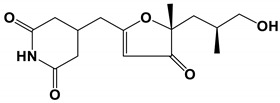	The MIC value was 4 μg/mL.	[83]
Funiculosone	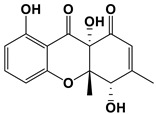	The IC_50_ value was 35 μg/mL.	[86]

BEC_80_: 80% of biofilm-eradicating concentration; MBIC: maximum biofilm inhibitory concentration; 5HM2F: 5-hydroxymethyl-2-furaldehyde.

## Data Availability

Not applicable.
